# An Exploratory Study of Host Plasma Proteomic Signatures that Distinguish Secondary and Early Latent Syphilis in Adults

**DOI:** 10.1093/ofid/ofag498

**Published:** 2026-08-04

**Authors:** Cody Chou, Shad R Morton, Kelika A Konda, Silver Vargas, Michael Reyes-Diaz, Francesca Vasquez, Carlos F Caceres, Jeffrey D Klausner, Trey Toombs, Rushdy Ahmad, Lao-Tzu Allan-Blitz

**Affiliations:** Department of Molecular and Cellular Biology, Harvard University, Boston, Massachusetts, USA; Wyss Diagnostics Accelerator, Wyss Institute, Boston, Massachusetts, USA; Department of Medicine and Population and Public Health Sciences, Keck School of Medicine, University of Southern California, Los Angeles, California, USA; Unit of Health, Sexuality and Human Development, and Laboratory of Sexual Health, Universidad Peruana Cayetano Heredia, Lima, Peru; Unit of Health, Sexuality and Human Development, and Laboratory of Sexual Health, Universidad Peruana Cayetano Heredia, Lima, Peru; Unit of Health, Sexuality and Human Development, and Laboratory of Sexual Health, Universidad Peruana Cayetano Heredia, Lima, Peru; Unit of Health, Sexuality and Human Development, and Laboratory of Sexual Health, Universidad Peruana Cayetano Heredia, Lima, Peru; Department of Medicine and Population and Public Health Sciences, Keck School of Medicine, University of Southern California, Los Angeles, California, USA; Wyss Diagnostics Accelerator, Wyss Institute, Boston, Massachusetts, USA; Wyss Diagnostics Accelerator, Wyss Institute, Boston, Massachusetts, USA; Division of Infectious Diseases, Department of Medicine, University of California Los Angeles, Los Angeles, California, USA; Division of Global Health Equity, Department of Medicine, Brigham and Women′s Hospital, Boston, MA, USA

**Keywords:** biomarkers, diagnostic development, proteomics, syphilis, *Treponema pallidum*

## Abstract

Syphilis diagnostics struggle to distinguish active from treated disease. Using tandem-mass-tag proteomics on plasma from 10 secondary and early latent syphilis cases and 8 matched controls, we identified 54 differentially regulated proteins categorized under several biological pathways. Those exploratory findings support proteomics as a tool for developing novel syphilis diagnostics.

In 2020, there were more than 8 million cases of syphilis, caused by *Treponema pallidum* infection [1]. Syphilis is diagnosed using treponemal and lipoidal (nontreponemal) antibody tests [2]. However, treponemal antibodies do not distinguish active from previously treated infection, and lipoidal test titers lack specificity [2]. Recent work identified differences in cytokine expression between individuals with syphilis and uninfected controls. We evaluated plasma protein profiles to identify additional host-response biomarkers that could distinguish individuals with secondary and early latent syphilis from those without the disease.

## METHODS

### Plasma Samples

We used plasma samples collected from patients recruited between May 2019 and June 2022 from the PICASSO study in Peru [3]. A PICASSO protocol amendment added a healthy control arm after syphilis case enrollment began. That allowed recruitment of participants without syphilis from the same clinics and with similar sociodemographic characteristics as the cases.

Clinicians diagnosed and staged syphilis according to the 2015 CDC guidelines using a rapid treponemal test—either determine syphilis TP (Abbott, United States) or *T. Pallidum* particle agglutination assays—as well as rapid plasma reagin (RPR) titers [4]. They classified patients as having secondary syphilis when they presented with consistent clinical manifestations and reactive treponemal and lipoidal tests, and early latent syphilis when they had reactive serology without symptoms of infection. We determined HIV-1/2 status for all participants using the Determine™ HIV-1/2 Ag/Ab Combo rapid test (Abbott, United States); however, we did not test participants for other sexually transmitted infections (STIs). Further, no data on concurrent inflammatory or autoimmune disease were available.

In the proteomic analyses, we included cases defined as adults with secondary or early latent syphilis, and controls defined as adults enrolled from the same clinics who had a nonreactive RPR and negative treponemal test. We selected plasma samples matched on age and HIV-1/2 status; however, males comprised a greater proportion of cases than controls and thus we could not match on gender. We collected samples at enrollment, prior to treatment. We analyzed secondary and early latent syphilis together to capture the spectrum of the host immune response along the continuum of early *T. pallidum* dissemination.

### Mass Spectrometry

We used tandem-mass-tag labeling to quantify peptide abundances across samples [5]. We depleted the 14 most abundant plasma proteins using Pierce Top 14 columns (Thermo Fisher Scientific, United States), then reduced the remaining proteins with tris (2-carboxyethyl) phosphine [C₉H₁₅O₆P], alkylated with iodoacetamide [C₂H₄INO], and digested them overnight with trypsin. We labeled the resulting peptides with the TMTpro 18-plex kit (Thermo Fisher Scientific), pooled, dried, and separated them into 18 fractions using high-pH reversed-phase chromatography.

We processed peptide spectra in Proteome Discoverer (Thermo Fisher Scientific) and used the Chimerys database to identify and quantify proteins. We normalized protein abundances to a pseudo-reference channel defined as the mean across samples. We imputed missing values using k-nearest neighbors and excluded proteins with more than 50% missingness. We assessed differential expression using limma in R version 4.5.0 (R Foundation, Austria) and performed feature selection and classification modeling in Python version 3.11.7 (Python Software Foundation, United States). We considered proteins differentially expressed if they demonstrated ≥1.5 absolute fold change and a Benjamini–Hochberg false discovery rate-adjusted *P*-value <.05.

We used the STRING database of known and predicted protein–protein associations for gene ontology and functional enrichment analyses. To characterize global expression patterns, we generated relative log expression plots and performed principal component analysis to assess separation between cases and controls. We then examined the functional significance of the observed proteomic alterations through protein–protein interaction network analyses.

We used a recursive feature elimination with cross-validation to identify a parsimonious subset of biomarkers predictive of syphilis. That involved training 4 machine-learning classification models while iteratively removing the least-associated feature. We calculated the area under the resulting receiver operating characteristics curves (AUC) and plotted confusion matrices using cross-validated predictions (scikit-learn) across 11 candidate models. We then selected the final model based on mean accuracy and lowest standard deviation across 1000 permutation tests.

### Ethical Considerations

The Universidad Cayetano Heredia Institutional Review Board approved the secondary use of stored samples. The Massachusetts General Brigham Institutional Review Board deemed the analysis of deidentified data did not constitute human subjects’ research.

## RESULTS

**Figure 1.**
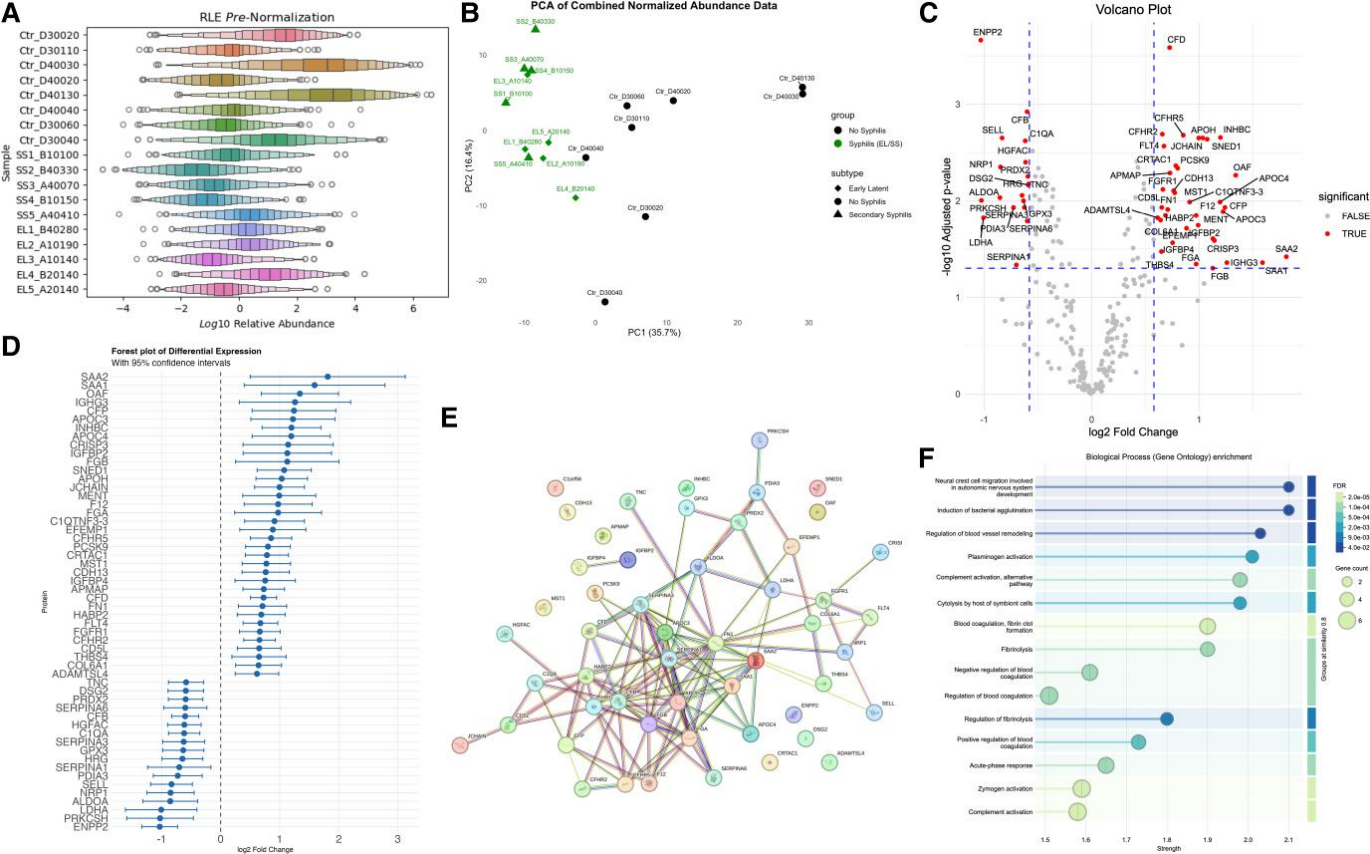
Primary proteomic differences between 10 cases of syphilis and 8 nondiseased controls. *A*, Relative Log Expression for all samples show heterogeneity in the control group (top 8), lower expression for secondary syphilis (middle) with early latent (bottom) slightly higher (RLE, relative log expression). *B*, principal component analysis captures the variability between the control and syphilis group, and the heterogeneity of the secondary and early latent groups (Ctr, control; SS, secondary syphilis; EL, early latent syphilis). *C*, Volcano plot showing significantly up and down regulated proteins; differences were considered significant when *P*-value <.05 (1.3 -log10) and log2 fold change > absolute value 0.58 (1.5-fold change). Case = Syphilis, Control = Nondiseased. *D*, Forest plot with 95% confidence interval of differentially expressed proteins ordered by fold change. *E*, Protein–protein interactions found by STRING database. *F*, Top-15 biological processes by strength shown with false discovery rate and protein count.

Baseline demographic and clinical characteristics of the 10 case-samples and 8 control-samples are shown in Table 1. Samples from controls exhibited upshifted median log10-relative peptide intensities of 0.899. Samples from cases with early latent syphilis exhibited median log10-relative peptide intensities values of 0.052, while samples from cases with secondary syphilis exhibited downshifted median log10-relative peptide intensities of −0.699 (Figure 1*A*). Principal component analyses demonstrated clear separation between syphilis cases and controls. The first principal component explained 35.7% of the total variance and separated controls from syphilis cases. The second principal component explained an additional 16.4% and distinguished secondary syphilis from early latent syphilis. Together, those accounted for 52.1% of the total variability (Figure 1*B*).

**Table 1.** Demographic and Serologic Characteristics of Individuals From Whom Samples Were Obtained for Proteomic Profiling

Characteristic	Cases (n = 10)	Controls (n = 8)
Median age at enrollment (IQR)	Median: 29.2 (23.6–31.9)	Median: 28.8 (26.6–35.9)
Sex		
Male	9 (90%)	5 (62.5%)
Female	1 (10%)	3 (37.5%)
HIV-1/2 Uninfected	10 (100%)	8 (100%)
Stage of syphilis		
Secondary syphilis	5 (50%)	0 (0%)
Early latent syphilis	5 (50%)	0 (0%)
Rapid plasma reagin (RPR) test result		
Reactive	10 (100%)	0 (0%)
Nonreactive	0 (0%)	8 (100%)
Treponemal test result		
Reactive	10 (100%)	0 (0%)

We identified 54 proteins that differed significantly between syphilis cases and controls, including 36 upregulated and 18 downregulated among cases (Figure 1*C* and 1*D*). Serum amyloid A2 (SAA2) (1.81 log2 fold change; 95% CI: 0.50–3.13) and serum amyloid A1 (SAA1) (1.59 log2 fold change; 95% CI: 0.40–2.78) were among the most upregulated proteins. Lactate Dehydrogenase A (−1.01 log2 fold change; 95% CI: −1.61 to −0.40) and Protein Kinase C Substrate 80K-H (PRKCSH) (−1.03 log2 fold change; 95% CI: −1.59 to −0.46) were among the most downregulated proteins. Figure 1*E* shows the protein–protein interactions found in the STRING database. Figure 1*F* shows the gene ontology associations. The Supplementary Table shows related functions of known protein signatures.

**Figure 2.**
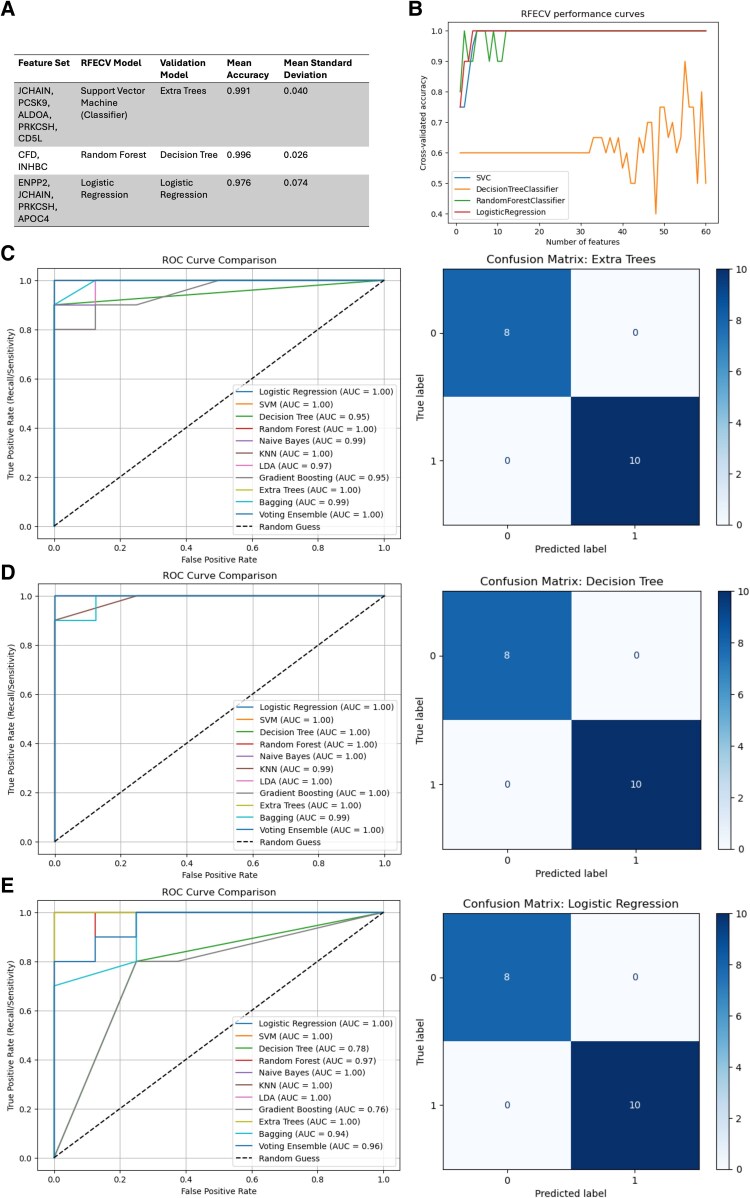
Identification of candidate protein signatures using 11 models with corresponding AUC plots and confusion matrices. *A*, Candidate proteins chosen by recursive feature elimination with cross-validation with permutation scores. (*B*) Recurrent feature elimination with cross-validation starting with all 54 features (+6 isoforms) (right), cross-validating by accuracy and eliminating one feature until none remain. *C*, Receiver operating characteristics curve and confusion matrix for first candidate protein panel (JCHAIN, PCSK9, ALDOA, PRKCSH, CD5L). *D*, Receiver operating characteristics curve and confusion matrix for second candidate protein panel (CFD, INHBC). *E*, Receiver operating characteristics curve and confusion matrix for third candidate protein panel (ENPP2, JCHAIN, PRKCSH, APOC4).

Recursive feature elimination with cross-validation produced a ranked feature list from the 54 candidate proteins, from which 9 were retained for evaluation across 3 panels (Figure 2*A*–2*E*). The 5-protein panel (JCHAIN, PCSK9, ALDOA, PRKCSH, CD5L; Figure 2*C*) achieved an AUC of 1.00 (95% CI: 1.00–1.00) using an Extra Trees classifier. The 2-protein panel (CFD, INHBC; Figure 2*D*) achieved an AUC of 0.996 (95% CI: 0.90–1.00) using a Decision Tree classifier. The 4-protein panel (ENPP2, JCHAIN, PRKCSH, APOC4; Figure 2*E*) achieved an AUC of 1.00 (95% CI: 1.00–1.00) using Logistic Regression.

## DISCUSSION

Protein signatures differed significantly between cases of secondary and early latent syphilis and controls. The 9 proteins most predictive of secondary and early latent syphilis originated from (1) immune and inflammatory responses (JCHAIN, CD5L, CFD, Serum Amyloid A1 and 2, and INHBC), (2) coagulation and vascular biology (PCSK9, APOC4, and ENPP2), and (3) cellular stress and damage responses (ALDOA and PRKCSH). Those pathways align with known features of *T. pallidum* infection and support the biological relevance of the proteomic shifts we observed [6, 7]. We found increased levels of FLT4, which encodes vascular endothelial growth factor receptor 3, typically expressed on lymphatic endothelium [8]. That is consistent with prior work demonstrating *T. pallidum* infection may trigger vascular responses [7]. JCHAIN transports immunoglobulins across mucosal epithelia and plays a role in humoral immunity [9]. SAA1 participates in host defense and inflammation by acting as an opsonin for certain bacteria, chemoattracting immune cells, and inducing enzymes that degrade the extracellular matrix [10]. During inflammation, SAA2 and SAA1 function as cytokine-like mediators [11]. Conversely, suppression of PRKCSH has been associated with enhanced antitumor immunity by impairing the maturation of regulatory glycoproteins on tumor cells [12]. In the context of infection, reduced PRKCSH expression might alter the presentation of glycosylated antigens. Earlier mass spectrometry studies focused largely on direct detection of *T. pallidum* derived peptides in serum or urine, an approach constrained by the low abundance of pathogen analytes in blood and by limited reproducibility across clinical samples [13, 14]. Studies of the host response, in turn, have relied predominantly on predefined cytokine panels measured by targeted immunoassays. In contrast, we performed untargeted profiling of the host plasma proteome. Reassuringly, our findings overlap with established biology, including acute-phase serum amyloid A and other inflammatory mediators, which supports their plausibility, while the untargeted design reveals additional candidates.

## LIMITATIONS

Our study had several limitations. First, the small sample size limits the precision of our findings, and the machine-learning analysis may render the model susceptible to overfitting despite cross-validation. Evaluating 11 classifiers across 54 candidate proteins in a dataset of 18 samples created a model selection bias and the high AUC values observed likely reflect that fact. Thus our results should be interpreted as hypothesis-generating. Further, because we evaluated thousands of proteins, some of the observed associations may reflect type I error. Importantly, the study lacked comparator samples from individuals with previously treated syphilis or other disease stages, as well as with concurrent STIs. Further, we did not have data on concurrent autoimmune or inflammatory conditions, which may confound proteomic profiling of host immune responses. Despite those limitations, our results indicate that proteomic analyses are promising strategies for furthering our understanding of the pathophysiology of *T. pallidum* infection and detection.

## CONCLUSION

In this pilot study, host plasma proteomic profiles distinguished adults with secondary and early latent syphilis from nondiseased controls. Our findings support further investigation of host-response proteomic biomarkers to advance our understanding of the disease and to evaluate their potential as tools to improve syphilis diagnosis.

## Supplementary Material

ofag498_Supplementary_Data

## Supplementary Data

Supplementary materials are available at *Open Forum Infectious Diseases* online. Consisting of data provided by the authors to benefit the reader, the posted materials are not copyedited and are the sole responsibility of the authors, so questions or comments should be addressed to the corresponding author.

## References

[1] WHO . Sexually Transmitted Infections (STIs). World Health Organization. Published May 29, 2025. Available at: https://www.who.int/news-room/fact-sheets/detail/sexually-transmitted-infections-(stis). Accessed March 10, 2026.

[2] Papp JR, Park IU, Fakile Y, Pereira L, Pillay A, Bolan GA. CDC laboratory recommendations for syphilis testing, United States, 2024. MMWR Recommendations and Reports 2024; 73:1–32. 10.15585/mmwr.rr7301a1 PMC10849099 38319847

[3] Reyes-Diaz M, Malca J, Konda KA, et al HIV infection modifies the role of prior Treponema pallidum infection in the clinical presentation of early syphilis among adult patients from sexually transmitted infection clinics in Peru. Sex Transm Dis 2024; 51:415–9. 38372543 10.1097/OLQ.0000000000001950 PMC11131579

[4] Workowski KA, Bolan GA; Centers for Disease Control and Prevention. Sexually transmitted diseases treatment guidelines, 2015. MMWR Recomm Rep 2015; 64(RR-03):1–137. PMC5885289 26042815

[5] Zecha J, Satpathy S, Kanashova T, et al TMT labeling for the masses: a robust and cost-efficient, in-solution labeling approach. Mol Cell Proteomics 2019; 18:1468–78. 30967486 10.1074/mcp.TIR119.001385 PMC6601210

[6] Liu A, Giacani L, Tuero I, Klausner JD. Syphilis pathogenesis: host immune response vs pathogen immune evasion. J Infect Dis 2025; 232:760–6. 40708500 10.1093/infdis/jiaf388 PMC12526937

[7] Waugh S, Ranasinghe A, Gomez A, et al Syphilis and the host: multi-omic analysis of host cellular responses to Treponema pallidum provides novel insight into syphilis pathogenesis. Front Microbiol 2023; 14:1254342. 37795301 10.3389/fmicb.2023.1254342 PMC10546344

[8] Lee JY, Lee S-E, Han A-R, Lee J, Yoon Y-S, Kim H-J. FLT4 as a marker for predicting prognostic risk of refractory acute myeloid leukemia. Haematologica 2023; 108:2933–45. 37317880 10.3324/haematol.2022.282472 PMC10620598

[9] Kawasaki K, Ohta Y, Castro CD, Flajnik MF. The immunoglobulin J chain is an evolutionarily co-opted chemokine. Proc Natl Acad Sci U S A 2024; 121:e2318995121. 38215184 10.1073/pnas.2318995121 PMC10801876

[10] Chang Y, Liu Y, Zou Y, Ye RD. Recent advances in studies of Serum amyloid A: implications in inflammation, immunity and tumor metastasis. Int J Mol Sci 2025; 26:987. 39940756 10.3390/ijms26030987 PMC11817213

[11] Cheng N, He R, Tian J, Ye PP, Ye RD. Cutting edge: TLR2 is a functional receptor for acute-phase serum amyloid A. J Immunol 2008; 181:22–6. 18566366 10.4049/jimmunol.181.1.22 PMC2464454

[12] Khaodee W, Xiyuan G, Han MTT, et al Transcriptomic analysis of glucosidase II beta subunit (GluIIß) knockout A549 cells reveals its roles in regulation of cell adhesion molecules (CAMs) and anti-tumor immunity. BMC Genomics 2024; 25:82. 38245670 10.1186/s12864-023-09888-z PMC10799456

[13] Van Raemdonck GA, Osbak KK, Van Ostade X, Kenyon CR. Needle lost in the haystack: multiple reaction monitoring fails to detect *Treponema pallidum* candidate protein biomarkers in plasma and urine samples from individuals with syphilis. F1000Res 2018; 7:336. 30519456 10.12688/f1000research.13964.1 PMC6248270

[14] Treger RS, Menza TW, Truong TT, Lieberman JA. Advances in syphilis diagnostics to address the 21st-century epidemic. Clin Chem 2025; 71:935–48. 40510004 10.1093/clinchem/hvaf072 PMC12405703

