# Supplementary material for: An Exploratory Study of Host Plasma Proteomic Signatures that Distinguish Secondary and Early Latent Syphilis in Adults
Source: Open Forum Infect Dis. 2026 Aug 4;13(8):ofag498. doi: 10.1093/ofid/ofag498 (PMC13481980; doi:10.1093/ofid/ofag498)
Supplement: Supplementary file 1 [file ofag498_supplementary_data.pdf]

## Supplementary Table: Biological Characterization of Up-Regulated and Down-Regulated Proteins.

| Up-Regulated Protein (Gene Name) | $Log_2$ Fold Change | Characterization of protein | Supplemental Citations |
|---|---|---|---|
| SAA2 | 1.81 | SAA2 is an acute-phase apolipoprotein produced primarily by hepatocytes in response to IL-1, IL-6, and TNF through NF-κB/STAT3 signaling. During inflammation, SAA2 (together with SAA1) can increase up to $10^3$-fold and functions as a cytokine-like mediator. It signals through TLR2, TLR4, and FPR2 to activate NF-κB, induce chemokine expression, and promote neutrophil and monocyte chemotaxis. At barrier sites, SAA2 also drives pathogenic Th17 cell differentiation. | 1, 2, 3 |
| SAA1 | 1.59 | SAA1 is an acute-phase protein produced by the liver during infection and tissue injury. It promotes leukocyte chemotaxis and Th17 polarization through FPR2 and TLR2/TLR4 signaling. | 4 |
| OAF | 1.34 | OAF is a member of the BRICHOS-domain protein family. BRICHOS domains function as intramolecular chaperones that prevent amyloid-like protein aggregation. | 5 |
| IGHG3 | 1.26 | IGHG3 encodes the constant region of the IgG3 heavy chain. Compared to other IgG subclasses, IgG3 has a relatively long hinge region that allows greater Fab arm flexibility. It is effective at complement activation, Fcγ receptor binding, | 6, 7 |

| | | | |
|---|---|---|---|
| | | opsonization, and pathogen clearance. IGHG3 is also highly polymorphic with numerous G3m allotypes contributing to considerable genetic diversity across individuals. | |
| CFP | 1.24 | CFP encodes properdin, which binds and stabilizes the alternative-pathway C3 convertase (C3bBb). It amplifies C3b deposition and downstream inflammation. Properdin can also act as a pattern-recognition molecule released locally by leukocytes to initiate alternative-pathway activity on microbial and apoptotic cell surfaces. | 8, 9 |
| APOC3 | 1.22 | APOC3 is an apolipoprotein produced by the liver and intestine that associates with triglyceride-rich lipoproteins. It inhibits lipoprotein lipase and hepatic uptake of triglyceride-rich lipoproteins. Beyond lipid metabolism, APOC3 can act as an endogenous damage-associated molecular pattern (DAMP) that triggers inflammation by activating the NLRP3 inflammasome in human monocytes through TLR2/4 and Syk/caspase-8 signaling which leads to IL-1β release and monocyte activation. | 10, 11 |
| INHBC | 1.2 | INHBC encodes a member of the TGF-β superfamily. Can form homodimers or heterodimers (with other beta-subunits) to function as activins/inhibins. Expression data shows that INHBC is expressed in immune-relevant cell types (e.g., monocytes & neutrophils) and lymphoid/bone-marrow tissues | 12, 13 |

| | | | |
|---|---|---|---|
| APOC4 | 1.2 | APOC4 belongs to the apolipoprotein C family, which regulates triglyceride-rich lipoprotein metabolism. It influences lipoprotein lipase activity and hepatic uptake of lipoproteins. | 14 |
| CRISP3 | 1.14 | CRISP3 is a secreted glycoprotein belonging to the CAP superfamily. CRISP3 is upregulated in sepsis, severe dengue, HCV infection, and neutrophil-activation gene clusters identified in long COVID. It is thought to contribute to neutrophil degranulation and modulation of inflammation. | 15, 16 |
| IGFBP2 | 1.13 | IGFBP2 binds IGF-I and IGF-II in circulation and modulates IGF receptor signaling. IGFBP2 can indirectly influence immune responses and inflammation. | 17 |
| FGB | 1.13 | FGB encodes the beta chain of fibrinogen. During vascular injury, thrombin cleaves fibrinopeptides A and B from fibrinogen and converts it to fibrin for blood clot formation. | 18 |
| SNED1 | 1.08 | SNED1 is a secreted protein that localizes to the extracellular matrix and mediates cell-matrix adhesion. Breast cancer cells and neural crest cells have been shown to adhere to SNED1 through its RGD motif via integrins $\alpha5\beta1$ and $\alpha v\beta3$. | 19 |
| APOH | 1.03 | APOH primarily functions as a coagulation factor and phospholipid-binding protein. It also acts as a scavenger of lipopolysaccharide (LPS) and certain pathogens. APOH is classified as an acute-phase protein and is upregulated during infection and inflammation. | 20 |

| | | | |
|---|---|---|---|
| JCHAIN | 1 | JCHAIN encodes the joining chain and promotes the assembly of IgA dimers and IgM pentamers. It enables binding to the polymeric immunoglobulin receptor (pIgR) on epithelial cells and facilitates trans-epithelial transport of secretory IgA and IgM into mucosal secretions. Expression of JCHAIN is largely restricted to B cells and plasma cells that produce polymeric IgA and IgM. | 21 |
| MENT | 0.99 | MENT is a member of the serpin (serine protease inhibitor) superfamily. It is a non-histone chromatin-associated protein that condenses chromatin into a more compact state and inhibits papain-family cysteine proteases. | 22 |
| F12 | 0.97 | F12 encodes the zymogen form of Factor XII. Once activated, Factor XIIa participates in the intrinsic coagulation cascade by activating Factor XI. | 23 |
| FGA | 0.97 | FGA encodes the alpha chain of fibrinogen which is a key coagulation protein | 24 |
| C1QTNF3-3 | 0.91 | C1QTNF3 (CTRP3) exerts anti-inflammatory and endothelial-protective effects. It dampens TLR4-driven NF-κB signaling, reduces endothelial expression of VCAM-1 and ICAM-1, decreases monocyte adhesion, and activates pro-survival PI3K/Akt/eNOS pathways. | 25, 26 |
| EFEMP1 | 0.88 | EFEMP1 encodes a secreted extracellular matrix glycoprotein. It interacts with tissue inhibitor of metalloproteinases-3 (TIMP-3) to modulate matrix metalloproteinase (MMP) activity and participates in cell-matrix | 27 |

| | | | |
|---|---|---|---|
| | | adhesion. | |
| CFHR5 | 0.85 | CFHR5 encodes a member of the Factor H-related (FHR) family of complement regulators. It binds complement C3 and is thought to modulate alternative pathway activity at sites of complement activation. | 28 |
| PCSK9 | 0.8 | PCSK9 is a regulator of cholesterol metabolism. It binds the low-density lipoprotein receptor (LDLR) and directs it for lysosomal degradation. Beyond lipid metabolism, PCSK9 can promote pro-inflammatory cytokine production and enhance NF-κB/TLR4-mediated signaling in macrophages and vascular cells. | 29, 30 |
| CRTAC1 | 0.78 | CRTAC1 encodes a cartilage-derived glycoprotein. It has been identified as a biomarker for osteoarthritis and lung adenocarcinoma. | 31 |
| MST1 | 0.77 | MST1 regulates T cell and B cell development and trafficking. It also participates in macrophage function, reactive oxygen species production, and inflammasome activation. | 32, 33 |
| CDH13 | 0.76 | CDH13 encodes a GPI-anchored cadherin that regulates GABAergic modulation in stem cell-derived neuronal networks. | 34 |
| IGFBP4 | 0.75 | IGFBP4 is a member of the insulin-like growth factor binding protein (IGFBP) family. It binds both IGF-I and IGF-II in circulation and modulates IGF bioavailability and receptor signaling | 35 |
| APMAP | 0.73 | APMAP localizes to the endoplasmic reticulum, where it suppresses lipid oxidation and supports lipoprotein processing. | 36 |

| | | | |
|---|---|---|---|
| | | These pathways can modulate innate immune responses in infected or inflamed tissues. | |
| CFD | 0.73 | CFD encodes a serine protease secreted primarily by adipocytes. It serves as a rate-limiting enzyme of the alternative complement pathway by cleaving factor B when complexed with C3b to generate the C3 convertase (C3bBb) and thus amplifies complement activation. | 37 |
| EN1 | 0.71 | EN1 has been linked to the fibroblast-to-myofibroblast transition and fibrotic disease. | 38 |
| HABP2 | 0.69 | HABP2 is a regulator of vascular endothelial barrier integrity. In models of acute lung injury, LPS stimulation increases HABP2 expression in pulmonary endothelial cells. Silencing HABP2 with small-interfering RNA attenuates LPS- and low-molecular-weight hyaluronan-induced endothelial hyperpermeability. HABP2 compromises vascular integrity through activation of a protease-activated receptor (PAR)-RhoA-Rho kinase signaling pathway. | 39 |
| FLT4 | 0.67 | FLT4 encodes a receptor tyrosine kinase that plays a role in lymphatic vessel formation and the maintenance of the lymphatic endothelium. | 40 |
| FGFR1 | 0.66 | FGFR1 encodes a receptor tyrosine kinase that binds fibroblast growth factor ligands and activates downstream signaling pathways that regulate cell proliferation and survival. | 41 |

| | | | |
|---|---|---|---|
| CFHR2 | 0.66 | CFHR2 is a regulator of the alternative complement pathway. It binds C3b and permits assembly of the C3 convertase but suppresses its catalytic activity and blocks formation of the terminal complement complex (TCC). | 42 |
| CD5L | 0.65 | CD5L encodes a soluble glycoprotein of the scavenger receptor cysteine-rich (SRCR) superfamily. It is predominantly produced and secreted by macrophages in lymphoid organs, liver, spleen, and inflamed tissues. Expression of CD5L is regulated by lipid-sensing nuclear receptors. | 43 |
| THBS4 | 0.65 | THBS4 encodes a thrombospondin family extracellular matrix glycoprotein. THBS4 participates in tissue remodeling and wound repair and contributes to vascular and ECM adaptive responses under stress. | 44 |
| COL6A1 | 0.64 | COL6A1 encodes the alpha-1 chain of type VI collagen which forms microfilament networks and contributes to cellular structural support. | 45 |
| ADAMTSL4 | 0.61 | ADAMTSL4 is a secreted protein that localizes to the extracellular matrix. It binds fibrillin-1 microfibrils and promotes microfibril assembly in tissue. | 46 |

| Down-Regulated Protein (Gene Name) | $Log_2$ Fold Change | Characterization of protein | Supplemental Citations |
|---|---|---|---|
| TNC | -0.59 | TNC is induced in tissues by pro-inflammatory stimuli and engages TLR4 to activate innate immune signaling and tissue | 47, 48 |

| | | | |
|---|---|---|---|
| | | repair. It modulates leukocyte behavior and contributes to inflammation during infection. | |
| DSG2 | -0.59 | DSG2 is essential for intestinal epithelial barrier integrity through its regulation of tight junction composition. Loss of DSG2 adhesion promotes inflammatory signaling and barrier dysfunction. | 49, 50, 51 |
| PRDX2 | -0.59 | PRDX2 functions as an intracellular antioxidant by catalyzing the reduction of hydrogen peroxide and organic hydroperoxides. Oxidized and glutathionylated PRDX2 can be released extracellularly and act as a damage-associated molecular pattern (DAMP). | 52 |
| SERPINA6 | -0.6 | SERPINA6 encodes corticosteroid-binding globulin (CBG) which is the primary plasma transport protein for glucocorticoids. It is produced mainly by the liver and circulates in plasma bound to cortisol. | 53 |
| CFB | -0.6 | CFB is a component of the alternative complement pathway. It enhances opsonization of pathogens through C3b deposition and facilitates phagocytosis. CFB also serves as a downstream effector of Toll-like receptor (TLR) signaling. Dysregulation of CFB has been implicated in complement-mediated diseases. | 54, 55 |
| HGFAC | -0.62 | HGFAC is primarily produced by hepatocytes and circulates in plasma as a zymogen. Its activation is associated to processes of tissue injury, coagulation and remodelling. | 56 |

| | | | |
|---|---|---|---|
| C1QA | -0.62 | C1QA encodes the A-chain polypeptide of complement subcomponent C1q. Together with the B and C chains, it forms the C1q molecule that binds antigen-antibody complexes and initiates cleavage of complement components C4 and C2. Mutations in C1QA are associated with autoimmunity and impaired clearance of apoptotic cells and immune complexes. | 57 |
| SERPINA3 | -0.63 | Elevated SERPINA3 levels have been associated with cerebral small-vessel disease severity. | 58 |
| GPX3 | -0.63 | GPX3 is a member of the glutathione peroxidase family. It is produced mainly by the kidneys and catalyzes the reduction of hydrogen peroxide and organic hydroperoxides using glutathione as a cofactor. | 59 |
| HRG | -0.65 | HRG encodes histidine-rich glycoprotein which is a plasma protein that modulate macrophage binding and coagulation. | 60 |
| SERPINA1 | -0.7 | SERPINA1 encodes alpha-1-antitrypsin which is a glycoprotein primarily synthesized by hepatocytes. It inhibits neutrophil elastase and other serine proteases to protect tissues from protease-mediated damage. | 61 |
| PDIA3 | -0.7 | PDIA3 encodes protein disulfide isomerase A3 which catalyzes disulfide bond formation through interactions with molecular chaperones. It plays a role in antigen presentation and is upregulated during infection. | 62 |

| | | | |
|---|---|---|---|
| SELL | -0.83 | SELL encodes L-selectin which is a cell-surface adhesion molecule of the selectin family expressed on most circulating leukocytes. It mediates the initial tethering and rolling of leukocytes on endothelial cells of high endothelial venules and inflamed microvessels. It facilitates leukocyte migration into tissues and lymphoid organs. | 63 |
| NRP1 | -0.85 | NRP1 encodes a transmembrane glycoprotein co-receptor expressed on neurons, endothelial cells, and various immune cell types. It contributes to immune regulation and cell migration. NRP1 has also been shown to regulate the stability and function of regulatory T cells. | 64 |
| ALDOA | -0.85 | ALDOA encodes aldolase A. | 65 |
| LDHA | -1.01 | LDHA encodes a subunit of lactate dehydrogenase. Elevated LDHA expression in tumors leads to lactate accumulation, which has been shown to suppress T cell function. | 66 |
| PRKCSH | -1.03 | PRKCSH encodes the beta subunit of glucosidase II which is an enzyme that resides in the endoplasmic reticulum and is involved in N-linked oligosaccharides and glycoprotein trafficking. In cancer, overexpression of PRKCSH has been associated with worse clinical outcomes. | 67 |
| ENPP2 | -1.03 | ENPP2 encodes a secreted lysophospholipase D that hydrolyzes lysophosphatidylcholine into lysophosphatidic acid (LPA). It plays a role in cell migration and has been associated with inflammation and cancer. | 68 |

Supplemental Citations


1. Chang Y, Liu Y, Zou Y, Ye RD. Recent Advances in Studies of Serum Amyloid A: Implications in Inflammation, Immunity and Tumor Metastasis. International Journal of Molecular Sciences. 2025; 26(3):987. https://doi.org/10.3390/ijms26030987
2. Ni Cheng, Rong He, Jun Tian, Patrick P Ye, Richard D Ye, Cutting Edge: TLR2 Is a Functional Receptor for Acute-Phase Serum Amyloid A, The Journal of Immunology, Volume 181, Issue 1, July 2008, Pages 22–26, https://doi.org/10.4049/jimmunol.181.1.22
3. Lee JY, Hall JA, Kroehling L, et al. Serum Amyloid A Proteins Induce Pathogenic Th17 Cells and Promote Inflammatory Disease. Cell. 2020;180(1):79-91.e16. doi:10.1016/j.cell.2019.11.026
4. Abouelasrar Salama S, De Bondt M, De Buck M, et al. Serum Amyloid A1 (SAA1) Revisited: Restricted Leukocyte-Activating Properties of Homogeneous SAA1. Front Immunol. 2020;11:843. Published 2020 May 14. doi:10.3389/fimmu.2020.00843
5. Sanchez-Pulido L, Ponting CP. OAF: a new member of the BRICHOS family. Bioinform Adv. 2022;2(1):vbac087. Published 2022 Nov 24. doi:10.1093/bioadv/vbac087
6. Damelang T, Rogerson SJ, Kent SJ, Chung AW. Role of IgG3 in Infectious Diseases. Trends Immunol. 2019;40(3):197-211. doi:10.1016/j.it.2019.01.005

7. Bashirova AA, Zheng W, Akdag M, et al. Population-specific diversity of the immunoglobulin constant heavy G chain (IGHG) genes. Genes Immun. 2021;22(7-8):327-334. doi:10.1038/s41435-021-00156-2
8. Hourcade DE. The role of properdin in the assembly of the alternative pathway C3 convertases of complement. J Biol Chem. 2006;281(4):2128-2132. doi:10.1074/jbc.M508928200
9. Cortes C, Ohtola JA, Saggu G, Ferreira VP. Local release of properdin in the cellular microenvironment: role in pattern recognition and amplification of the alternative pathway of complement. Front Immunol. 2013;3:412. Published 2013 Jan 17. doi:10.3389/fimmu.2012.00412
10. Norata GD, Tsimikas S, Pirillo A, Catapano AL. Apolipoprotein C-III: From Pathophysiology to Pharmacology. Trends Pharmacol Sci. 2015;36(10):675-687. doi:10.1016/j.tips.2015.07.001
11. Zewinger S, Reiser J, Jankowski V, et al. Apolipoprotein C3 induces inflammation and organ damage by alternative inflammasome activation. Nat Immunol. 2020;21(1):30-41. doi:10.1038/s41590-019-0548-1
12. LinkedOmics KB [Internet]. INHBC gene. Linkedomics.org; c2026. https://kb.linkedomics.org/gene/INHBC. Accessed February 23, 2026.
13. The Human Protein Atlas [Internet]. INHBC gene summary. Stockholm, Sweden: The Human Protein Atlas; c2026. https://www.proteinatlas.org/ENSG00000175189-INHBC/summary/gene. Accessed February 23, 2026.

14. Jong MC, Hofker MH, Havekes LM. Role of ApoCs in lipoprotein metabolism: functional differences between ApoC1, ApoC2, and ApoC3. Arterioscler Thromb Vasc Biol. 1999;19(3):472-484. doi:10.1161/01.atv.19.3.472
15. Anklesaria JH, Pandya RR, Pathak BR, Mahale SD. Purification and characterization of CRISP-3 from human seminal plasma and its real-time binding kinetics with PSP94. J Chromatogr B Analyt Technol Biomed Life Sci. 2016;1039:59-65. doi:10.1016/j.jchromb.2016.10.032
16. Zhang A-q, Wen D-l, Ma X-x, Zhang F, Chen G-s, Maimaiti K, Xu G, Jiang J-x and Lu H-x (2024) Upregulation of CRISP3 and its clinical values in adult sepsis: a comprehensive analysis based on microarrays and a two-retrospective-cohort study. Front. Immunol. 15:1492538. doi: 10.3389/fimmu.2024.1492538
17. Huynh H, Zheng J, Umikawa M, et al. IGF binding protein 2 supports the survival and cycling of hematopoietic stem cells. Blood. 2011;118(12):3236-3243. doi:10.1182/blood-2011-01-331876
18. Wolberg AS. Fibrinogen and fibrin: synthesis, structure, and function in health and disease. J Thromb Haemost. 2023;21(11):3005-3015. doi:10.1016/j.jtha.2023.08.014
19. Pally D, Kapoor N, Naba A. The novel ECM protein SNED1 mediates cell adhesion via the RGD-binding integrins α5β1 and αvβ3. Preprint. bioRxiv. 2024;2024.08.07.606706. Published 2024 Nov 21. doi:10.1101/2024.08.07.606706
20. Sorgenfrei M, Hürlimann LM, Remy MM, Keller PM, Seeger MA. Biomolecules capturing live bacteria from clinical samples. Trends Biochem Sci. 2022;47(8):673-688. doi:10.1016/j.tibs.2022.03.018

21. Kawasaki K, Ohta Y, Castro CD, Flajnik MF. The immunoglobulin J chain is an evolutionarily co-opted chemokine. Proc Natl Acad Sci U S A. 2024;121(3):e2318995121. doi:10.1073/pnas.2318995121
22. Irving JA, Shushanov SS, Pike RN, et al. Inhibitory activity of a heterochromatin-associated serpin (MENT) against papain-like cysteine proteinases affects chromatin structure and blocks cell proliferation. J Biol Chem. 2002;277(15):13192-13201. doi:10.1074/jbc.M108460200
23. Nickel KF, Long AT, Fuchs TA, Butler LM, Renné T. Factor XII as a Therapeutic Target in Thromboembolic and Inflammatory Diseases. Arterioscler Thromb Vasc Biol. 2017;37(1):13-20. doi:10.1161/ATVBAHA.116.308595
24. Cheung EY, Bos MJ, Leebeek FW, et al. Variation in fibrinogen FGG and FGA genes and risk of stroke: the Rotterdam Study. Thromb Haemost. 2008;100(2):308-313.
25. Guo S, Mao X and Liu J (2023) Multi-faceted roles of C1q/TNF-related proteins family in atherosclerosis. Front. Immunol. 14:1253433. doi: 10.3389/fimmu.2023.1253433
26. Schmid A, Vlacil A-K, Schuett J, Karrasch T, Schieffer B, Schäffler A, Grote K. Anti-Inflammatory Effects of C1q/Tumor Necrosis Factor-Related Protein 3 (CTRP3) in Endothelial Cells. Cells. 2021; 10(8):2146. https://doi.org/10.3390/cells10082146
27. Wood, A. J., Livingstone, I., Westcott, M., Furniss, D., & Wiberg, A. (2025). A review of the role of EFEMP1 in ophthalmic disease. Ophthalmic Genetics, 46(6), 523–531. https://doi.org/10.1080/13816810.2025.2524511

28. Jennifer L McRae, Thomas G Duthy, Kim M Griggs, Rebecca J Ormsby, Peter J Cowan, Brett A Cromer, William J McKinstry, Michael W Parker, Brendan F Murphy, David L Gordon, Human Factor H-Related Protein 5 Has Cofactor Activity, Inhibits C3 Convertase Activity, Binds Heparin and C-Reactive Protein, and Associates with Lipoprotein, The Journal of Immunology, Volume 174, Issue 10, May 2005, Pages 6250–6256, https://doi.org/10.4049/jimmunol.174.10.6250
29. Nabil G Seidah, Annik Prat, The Multifaceted Biology of PCSK9, Endocrine Reviews, Volume 43, Issue 3, June 2022, Pages 558–582, https://doi.org/10.1210/endrev/bnab035
30. Zufeng Ding, Naga Venkata K Pothineni, Akshay Goel, Thomas F Lüscher, Jawahar L Mehta, PCSK9 and inflammation: role of shear stress, pro-inflammatory cytokines, and LOX-1, Cardiovascular Research, Volume 116, Issue 5, 1 April 2020, Pages 908–915, https://doi.org/10.1093/cvr/cvz313
31. Tan, L., Zhang, H., Ding, Y. et al. CRTAC1 identified as a promising diagnosis and prognostic biomarker in lung adenocarcinoma. Sci Rep 14, 11223 (2024). https://doi.org/10.1038/s41598-024-61804-x
32. Qi Y, Sun D, Yang W, et al. Mammalian Sterile 20-Like Kinase (MST) 1/2: Crucial Players in Nervous and Immune System and Neurological Disorders. J Mol Biol. 2020;432(10):3177-3190. doi:10.1016/j.jmb.2020.03.010
33. Ueda Y, Kondo N, Kinashi T. MST1/2 Balance Immune Activation and Tolerance by Orchestrating Adhesion, Transcription, and Organelle Dynamics in Lymphocytes. Front Immunol. 2020;11:733. Published 2020 May 6. doi:10.3389/fimmu.2020.00733

34. Mossink, B., van Rhijn, JR., Wang, S. et al. Cadherin-13 is a critical regulator of GABAergic modulation in human stem-cell-derived neuronal networks. Mol Psychiatry 27, 1–18 (2022). https://doi.org/10.1038/s41380-021-01117-x
35. Hjortebjerg R. IGFBP-4 and PAPP-A in normal physiology and disease. Growth Horm IGF Res. 2018;41:7-22. doi:10.1016/j.ghir.2018.05.002
36. Paul B, Merta H, Ugrankar-Banerjee R, et al. Paraoxonase-like APMAP maintains endoplasmic-reticulum-associated lipid and lipoprotein homeostasis. Dev Cell. 2025; Published online May 2, 2025. doi:10.1016/j.devcel.2025.04.008
37. Kong Y, Wang N, Tong Z, Wang D, Wang P, Yang Q, Yan X, Song W, Jin Z and Zhang M (2024) Role of complement factor D in cardiovascular and metabolic diseases. Front. Immunol. 15:1453030. doi: 10.3389/fimmu.2024.1453030
38. Györfi AH, Matei AE, Fuchs M, et al. Engrailed 1 coordinates cytoskeletal reorganization to induce myofibroblast differentiation. J Exp Med. 2021;218(9):e20201916. doi:10.1084/jem.20201916
39. Mambetsariev N, Mirzapoiazova T, Mambetsariev B, et al. Hyaluronic Acid binding protein 2 is a novel regulator of vascular integrity. Arterioscler Thromb Vasc Biol. 2010;30(3):483-490. doi:10.1161/ATVBAHA.109.200451
40. Richard M Monaghan, Richard W Naylor, Daisy Flatman, Paul R Kasher, Simon G Williams, Bernard D Keavney, FLT4 causes developmental disorders of the cardiovascular and lymphovascular systems via pleiotropic molecular mechanisms, Cardiovascular Research, Volume 120, Issue 10, July 2024, Pages 1164–1176, https://doi.org/10.1093/cvr/cvae104

41. Xie Y, Su N, Yang J, et al. FGF/FGFR signaling in health and disease. Signal Transduct Target Ther. 2020;5(1):181. Published 2020 Sep 2. doi:10.1038/s41392-020-00222-7
42. Eberhardt HU, Buhlmann D, Hortschansky P, et al. Human factor H-related protein 2 (CFHR2) regulates complement activation. Plos one. 2013 ;8(11):e78617. DOI: 10.1371/journal.pone.0078617. PMID: 24260121; PMCID: PMC3832495.
43. Sanchez-Moral L, Ràfols N, Martori C, Paul T, Téllez É, Sarrias M-R. Multifaceted Roles of CD5L in Infectious and Sterile Inflammation. International Journal of Molecular Sciences. 2021; 22(8):4076. https://doi.org/10.3390/ijms22084076
44. Klaas M, Mäemets-Allas K, Heinmäe E, Lagus H, Cárdenas-León CG, Arak T, Eller M, Kingo K, Kankuri E and Jaks V (2021) Thrombospondin-4 Is a Soluble Dermal Inflammatory Signal That Selectively Promotes Fibroblast Migration and Keratinocyte Proliferation for Skin Regeneration and Wound Healing. Front. Cell Dev. Biol. 9:745637. doi: 10.3389/fcell.2021.745637
45. MedlinePlus [Internet]. Bethesda (MD): National Library of Medicine (US); [updated 2020 Jun 24]. COL6A1 gene; [updated 2022 Jul 19; reviewed 2022 Jul 19; cited 2026 Feb 23]; [about 5 p.]. Available from: https://medlineplus.gov/genetics/gene/col6a1/
46. MedlinePlus [Internet]. Bethesda (MD): National Library of Medicine (US); [updated Mar 1, 2015]. ADAMTSL4 gene; [cited 2026 Feb 23]. Available from: https://medlineplus.gov/genetics/gene/adamtsl4/
47. Meijer MT, de Vos AF, Scicluna BP, et al. Tenascin-C Deficiency Is Associated With Reduced Bacterial Outgrowth During Klebsiella pneumoniae-Evoked

Pneumosepsis in Mice. Front Immunol. 2021;12:600979. Published 2021 Mar 11. doi:10.3389/fimmu.2021.600979

48. Yilmaz A, Loustau T, Salomé N, et al. Advances on the roles of tenascin-C in cancer. J Cell Sci. 2022;135(18):jcs260244. doi:10.1242/jcs.260244

49. Yulis M, Quiros M, Hilgarth R, Parkos CA, Nusrat A. Intracellular Desmoglein-2 cleavage sensitizes epithelial cells to apoptosis in response to pro-inflammatory cytokines. Cell Death Dis. 2018;9(3):389. Published 2018 Mar 9. doi:10.1038/s41419-018-0380-9

50. Burkard N, Meir M, Kannapin F, Otto C, Petzke M, Germer C-T, Waschke J and Schlegel N (2021) Desmoglein2 Regulates Claudin2 Expression by Sequestering PI-3-Kinase in Intestinal Epithelial Cells. Front. Immunol. 12:756321. doi: 10.3389/fimmu.2021.756321

51. Gross A, Pack LAP, Schacht GM, et al. Desmoglein 2, but not desmocollin 2, protects intestinal epithelia from injury. Mucosal Immunol. 2018;11(6):1630-1639. doi:10.1038/s41385-018-0062-z

52. Salzano S, Checconi P, Hanschmann EM, et al. Linkage of inflammation and oxidative stress via release of glutathionylated peroxiredoxin-2, which acts as a danger signal. Proc Natl Acad Sci U S A. 2014;111(33):12157-12162. doi:10.1073/pnas.1401712111

53. Crawford, A.A., Bankier, S., Altmaier, E. et al. Variation in the SERPINA6/SERPINA1 locus alters morning plasma cortisol, hepatic corticosteroid binding globulin expression, gene expression in peripheral tissues, and risk of cardiovascular disease. J Hum Genet 66, 625–636 (2021). https://doi.org/10.1038/s10038-020-00895-6

54. Lin Zou, Yan Feng, Yan Li, Ming Zhang, Chan Chen, Jiayan Cai, Yu Gong, Larry Wang, Joshua M Thurman, Xiaobo Wu, John P Atkinson, Wei Chao, Complement Factor B Is the Downstream Effector of TLRs and Plays an Important Role in a Mouse Model of Severe Sepsis, The Journal of Immunology, Volume 191, Issue 11, December 2013, Pages 5625–5635, https://doi.org/10.4049/jimmunol.1301903
55. Kavanagh D, Barratt J, Schubart A, Webb NJA, Meier M and Fakhouri F (2025) Factor B as a therapeutic target for the treatment of complement-mediated diseases. Front. Immunol. 16:1537974. doi: 10.3389/fimmu.2025.1537974
56. Fukushima T, Uchiyama S, Tanaka H, Kataoka H. Hepatocyte Growth Factor Activator: A Proteinase Linking Tissue Injury with Repair. International Journal of Molecular Sciences. 2018; 19(11):3435. https://doi.org/10.3390/ijms19113435
57. Ghebrehiwet B, Kandov E, Kishore U, Peerschke EIB. Is the A-Chain the Engine That Drives the Diversity of C1q Functions? Revisiting Its Unique Structure. Front Immunol. 2018;9:162. Published 2018 Feb 5. doi:10.3389/fimmu.2018.00162
58. Hu X, Xiao ZS, Shen YQ, et al. SERPINA3: A novel inflammatory biomarker associated with cerebral small vessel disease burden in ischemic stroke. CNS Neurosci Ther. 2024;30(3):e14472. doi:10.1111/cns.14472
59. Zhang N, Liao H, Lin Z, Tang Q. Insights into the Role of Glutathione Peroxidase 3 in Non-Neoplastic Diseases. Biomolecules. 2024; 14(6):689. https://doi.org/10.3390/biom14060689
60. Poon IK, Patel KK, Davis DS, Parish CR, Hulett MD. Histidine-rich glycoprotein: the Swiss Army knife of mammalian plasma. Blood. 2011;117(7):2093-2101. doi:10.1182/blood-2010-09-303842

61. Mazzuca C, Vitiello L, Travaglini S, Maurizi F, Finamore P, Santangelo S, Rigon A, Vadacca M, Angeletti S and Scarlata S (2024) Immunological and homeostatic pathways of alpha -1 antitrypsin: a new therapeutic potential. Front. Immunol. 15:1443297. doi: 10.3389/fimmu.2024.1443297
62. Chichiarelli, S., Altieri, F., Paglia, G. et al. ERp57/PDIA3: new insight. Cell Mol Biol Lett 27, 12 (2022). https://doi.org/10.1186/s11658-022-00315-x
63. Ivetic A, Hoskins Green HL and Hart SJ (2019) L-selectin: A Major Regulator of Leukocyte Adhesion, Migration and Signaling. Front. Immunol. 10:1068. doi: 10.3389/fimmu.2019.01068
64. Roy S, Bag AK, Singh RK, Talmadge JE, Batra SK and Datta K (2017) Multifaceted Role of Neuropilins in the Immune System: Potential Targets for Immunotherapy. Front. Immunol. 8:1228. doi: 10.3389/fimmu.2017.01228
65. Nascentes Melo, L.M., Cansiz, F. & Tasdogan, A. Aldolase A: the broker of glycolysis. Nat Metab 7, 242–244 (2025). https://doi.org/10.1038/s42255-024-01202-9
66. Brand A, Singer K, Koehl GE, et al. LDHA-Associated Lactic Acid Production Blunts Tumor Immunosurveillance by T and NK Cells. Cell Metab. 2016;24(5):657-671. doi:10.1016/j.cmet.2016.08.011
67. Cressey R, Han MTT, Khaodee W, Xiyuan G, Qing Y. Navigating PRKCSH's impact on cancer: from N-linked glycosylation to death pathway and anti-tumor immunity. Front Oncol. 2024;14:1378694. Published 2024 Mar 20. doi:10.3389/fonc.2024.1378694
68. Cholia RP, Nayyar H, Kumar R, Mantha AK. Understanding the Multifaceted Role of Ectonucleotide Pyrophosphatase/Phosphodiesterase 2 (ENPP2) and its

Altered Behaviour in Human Diseases. Curr Mol Med. 2015;15(10):932-943. doi:10.2174/1566524015666150921104804
